# Short desynchronization epochs in neural synchronization: detection, mechanisms, and functions

**DOI:** 10.1186/1471-2202-16-S1-P3

**Published:** 2015-12-18

**Authors:** Leonid L Rubchinsky, Sungwoo Ahn

**Affiliations:** 1 Department of Mathematical Sciences, Indiana University Purdue University Indianapolis, Indianapolis, IN, USA; 2 Stark Neurosciences Research Institute, Indiana University School of Medicine, Indianapolis, IN, USA; 3 Present address: School of Mathematical and Statistical Sciences, Arizona State University, Tempe, AZ, USA

## 

Synchrony of neural oscillations is believed to play important role in a variety of functions and dysfunctions of neural systems. However, neural synchrony can hardly be perfect for prolong intervals of time. If there is a significant synchrony strength observed over sufficiently long time interval, the imperfect nature of synchrony implies that for some intervals of time synchrony may be stronger, while for some intervals of time in may be weaker. If neural synchrony is to play some functional role, this role may depend on how synchrony is patterned in time. Roughly speaking, few long intervals of synchronous (or nonsynchronous) dynamics may be functionally different from many short intervals, although the synchrony strength may be the same on the average.

Recent developments in time-series analysis allowed exploring the patterning of synchrony in time on very short time-scales. If there is some synchrony level is present on the average, than one can look at each cycle of oscillations and detect of signals are synchronous or not resulting in a quantitative description of succession of episodes of synchronization and desynchronization [1]. We applied these methods to analyze neural signals of various natures: synchronization of single units and LFPs in subthalamic nucleus of Parkinsonian patients [2], synchronization of EEG signals in healthy humans [3], synchronization of LFPs within and between prefrontal and hippocampal circuits in normal rats and rats experiencing repetitive psychostimulant injections [4]). The temporal patterning of the neural synchrony was sensitive to behavioral states, drug status etc. However, all these studies yielded one important qualitative results: neural oscillations were observed to go out of synchrony primarily for a short time interval. They may go out of synch very frequently (leading to low synchrony levels), but all these different neural systems exhibit mostly very short desynchronization intervals (different from rare prolongs desynchronizations and a spectrum of possibilities in between, none of which were experimentally observed). On the contrary, non-neural systems can exhibit longer desynchronization durations [1,5].

These experimental results suggest that short desynchronization dynamics may be universal for synchronized activity of neural systems. Here we review the data analysis techniques and results of their application to experimental data. We then consider the potential mechanisms of the apparent universality of short desynchronization dynamics and its potential functional advantages. We consider simple neural circuits of Morris-Lecar-type neurons and identify conditions imposed on the parameter values to result in both short desynchronization dynamics and dynamics exhibiting longer and rarer desynchronized intervals. By maintaining the same levels of average synchrony (and the same frequency of oscillations) we show that fast conductances promote short desynchronization dynamics observed in experiments. By subjecting these model networks to oscillatory synaptic input, we found that systems with short desynchronization dynamics require weaker input signal in order to synchronize with this input. We finally conjecture that properties of ionic conductances responsible for short desynchronization dynamics may make neural systems more adaptable, as they are more efficient in generating transient synchronous patterns (synchronous assemblies) in response to synaptic or sensory inputs.
